# Environments that Induce Synthetic Microbial Ecosystems

**DOI:** 10.1371/journal.pcbi.1001002

**Published:** 2010-11-18

**Authors:** Niels Klitgord, Daniel Segrè

**Affiliations:** 1 Bioinformatics Program, Boston University, Boston, Massachusetts, United States of America; 2 Department of Biology and Department of Biomedical Engineering, Boston University, Boston, Massachusetts, United States of America; University of Virginia, United States of America

## Abstract

Interactions between microbial species are sometimes mediated by the exchange of small molecules, secreted by one species and metabolized by another. Both one-way (commensal) and two-way (mutualistic) interactions may contribute to complex networks of interdependencies. Understanding these interactions constitutes an open challenge in microbial ecology, with applications ranging from the human microbiome to environmental sustainability. In parallel to natural communities, it is possible to explore interactions in artificial microbial ecosystems, e.g. pairs of genetically engineered mutualistic strains. Here we computationally generate artificial microbial ecosystems without re-engineering the microbes themselves, but rather by predicting their growth on appropriately designed media. We use genome-scale stoichiometric models of metabolism to identify media that can sustain growth for a pair of species, but fail to do so for one or both individual species, thereby inducing putative symbiotic interactions. We first tested our approach on two previously studied mutualistic pairs, and on a pair of highly curated model organisms, showing that our algorithms successfully recapitulate known interactions, robustly predict new ones, and provide novel insight on exchanged molecules. We then applied our method to all possible pairs of seven microbial species, and found that it is always possible to identify putative media that induce commensalism or mutualism. Our analysis also suggests that symbiotic interactions may arise more readily through environmental fluctuations than genetic modifications. We envision that our approach will help generate microbe-microbe interaction maps useful for understanding microbial consortia dynamics and evolution, and for exploring the full potential of natural metabolic pathways for metabolic engineering applications.

## Author Summary

Microbial metabolism affects biogeochemical cycles and human health. In most natural environments, multiple microbial species interact with each other, forming complex ecosystems whose properties are poorly understood. In an effort to understand inter-microbial interactions, and to explore new metabolic engineering avenues, researchers have started building artificial microbial ecosystems, e.g. pairs of genetically engineered strains that require each other for survival. Here we computationally explore the possibility of creating artificial microbial ecosystems without re-engineering the microbes themselves, but rather by manipulating the environment in which they grow. Specifically, using the framework of flux balance analysis, we predict environments in which either one or both microbes in a pair would not be able to grow without the other, inducing commensal (one-way) or mutualistic (two-way) interactions, respectively. Our algorithms can successfully recapitulate known inter-microbial interactions, and predict millions of new ones across any pair amongst different microbial species. Surprisingly, we find that it is always possible to identify conditions that induce mutualistic or commensal interactions between any two species. Hence, our method should help in mapping naturally occurring microbe-microbe interactions, and in engineering new ones through a novel, environment-driven branch of synthetic ecology.

## Introduction

While several aspects of microbial metabolism can be fruitfully addressed by studying individual microbial species, many contemporary challenges, including environmental remediation and infectious diseases, require a massive effort towards understanding how microbes interact with each other. In fact, in nature, most microbes do not live in isolation, but rather exist as part of complex, dynamically changing, microbial consortia [1], [2]. From a metabolic perspective, the coordinated action of multiple interacting microbes is known to enable specific metabolic processes, such as the bio-geochemical process of nitrification that occurs in soil and marine water [3], pesticide degradation in agricultural settings [4], anaerobic methanogenesis in animal rumen, fresh water ponds and sewage sludge digester [5], anaerobic oxidation of methane in marine environments [6] or degradation of xylan or complex oligosaccharides in the microbial flora of the human gut [7], [8]. Metabolic interdependencies are also thought to partially be associated with the problem of microbial unculturability [9].

**Figure 1:**
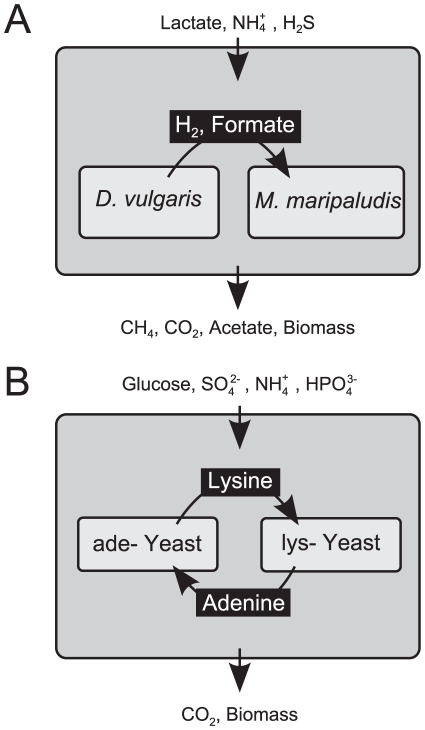
Two known examples of metabolism-based symbiotic interactions which we use as test cases for our algorithms. Chemical formulas represent metabolites and arrows represent possible exchange or transport reactions. **A** Experimental and computational setup of the methanogenic community composed of *Desulfovibrio vulgaris* and *Methanococcus maripaludis* [32]. *D. vulgaris* consumes lactate and produces formate and hydrogen which are consumed by *M. maripaludis*. While this may seem a one-way, i.e. commensal interaction, it has been determined that *D. vulgaris* benefits from hydrogen consumption by *M. maripaludis*, as this reduces the hydrogen partial pressure, allowing *D. vulgaris* to continue its metabolic processes. Hence, this should be considered a case of mutualistic interaction. **B** Schematic representation of the yeast synthetic ecosystem experimentally constructed in [19]. Yeast strains Ade- and Lys- where engineered to be auxotrophic for adenine and lysine respectively. It has been shown that neither yeast strain could grow on a minimal glucose medium alone. The same medium however does allow both to grow syntrophically in co-culture, demonstrating a mutualistic interaction.

Metabolic interactions between pairs of microbial species could be thought of as unidirectional or bidirectional exchanges of small molecules, which may benefit one or both species (Table 1). A commensal interaction is a one-way exchange, where one organism is dependent on the product of the other. An obligate bidirectional exchange (commonly referred to as cross-feeding, syntrophy or mutualism) is perhaps the most fascinating of all possible interactions. Such an interaction implies a mutual dependence, which seems contingent on the rise of improbable matching of resource requirements and availabilities. Metabolic syntrophy is thought to drive fundamental biogeochemical processes (Fig. 1, [10]–[13]), either through the mutual benefit of a uni-directional nutrient exchange (Fig. 1A), or through bi-directional cross-feeding [8], [10], [11], [14], [15]. In addition, engineered species can be induced to display mutualistic interactions, as shown in classical work aimed at unraveling the order of metabolic reactions in biosynthetic pathways [16]–[18], and in recent synthetic ecology experiments [19]–[21] (Fig. 1B).

**Table 1:** Definition and description of possible types of interactions, as used in the current work and described in the literature.

Interaction	Definition used in this work	Notes
Mutualism	Obligatory cross-feeding of metabolites. Each organism provides a metabolite essential to the other organism	This is also sometimes called syntrophy or symbiosis
Commensalism	One organism depends on the other for supply of a nutrient essential for growth	If the organism supplying a metabolite to the other pays a fitness price, this is known as parasitism
Neutralism	Neither organism depends on cross-feeding for growth	Since the two species are sharing the same resources, this situation could give rise to competition

In parallel to experimental studies, the rise of genome-scale constraint-based models of metabolism has the potential to help address questions that cannot be easily addressed experimentally. Constraint-based models of metabolic networks represent an efficient framework for a quantitative understanding of microbial physiology [22] (see Methods). Such models rely on the knowledge of the stoichiometry for every known metabolic reaction taking place in the cell, and focus on predicting steady state fluxes (i.e. reaction rates) rather than time-dependent metabolite concentrations. By focusing on the fluxes, one can view cellular metabolism as a resource allocation problem: given that the system has internal stoichiometric and thermodynamic constraints, and a certain amount of nutrients available, how should the flow through the network be distributed to allow the cell to achieve a given biological task, e.g. grow at maximal possible rate? This approach, also known as flux balance analysis, has been described in detail elsewhere [23]–[26], and given rise to a plethora of interesting discussions on optimality in metabolic network regulation and evolution [23], [27]–[30]. In the study of microbial ecosystems, it has been recognized that the extension of constraint-based models from individual to multiple interacting species or compartments involves novel challenges and opportunities [31]–[33]. In particular it has been shown that stoichiometric models of individual species can be combined to provide testable predictions about ecosystem-level behavior [32], [33]. The alternative method of network expansion has been used to identify putative metabolic synergy between all pairs of nearly 450 organisms in a single environmental setting [34]. Moreover, in broader context, evolutionary and functional insight was obtained through large meta-metabolism models that ignore the spatial distinctions between different organisms [35]–[37].

Here, we use constraint-based models to develop a new strategy for the study of metabolism-based symbiotic interactions in pairs of microbial species. While in most analyses of cross-feeding interactions the focus is on the properties of the organisms themselves, we take a different approach, asking whether, given two arbitrary organisms, it is possible to identify environmental conditions that induce a mutualistic or commensal interaction. We start by exploring known symbiotic pairs, to determine if available stoichiometric models seem to provide predictions that are in agreement with empirical observations. The algorithms we developed allow us not only to verify potential interactions, but also to produce lists of putatively exchanged metabolites. Then, we ask whether, given any two species whose stoichiometric models are available, it is possible to predict potential nutrient compositions that induce specific symbiotic behaviors, in particular commensalism and mutualism. Hence, taking advantage of the efficiency of constraint-based models, we explore the large space of possible media compositions, in search for nutrient combinations that sustain a co-culture of two species but do not support growth of each organism on its own. We apply our pipeline to the prediction of novel environments and interactions for a coculture of *Escherichia coli* and *Saccharomyces cerevisiae*, and for all pairwise combinations of seven bacterial species: *Escherichia coli*, *Helicobacter pylori*, *Salmonella typhimurium*, *Bacillus subtilis*, *Shewanella oneidensis*, *Methylobacterium extorquens*, *and Methanosarcina barkeri*. In addition to providing an algorithmic platform for synthetic ecology exploration, we envisage that our approach will help mapping and understanding interactions that occur in natural microbial consortia.

## Results

**Figure 2:**
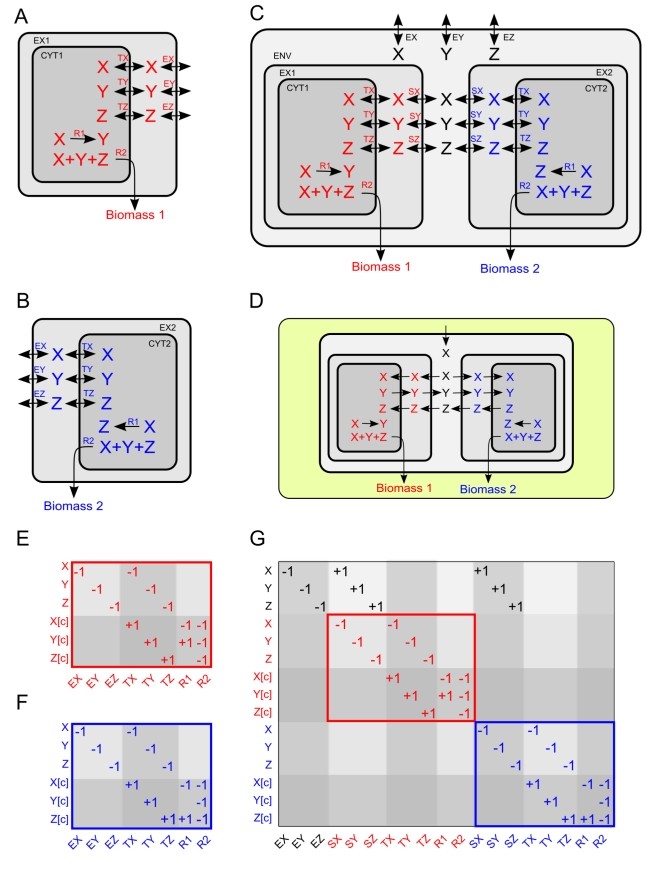
Construction of a joint metabolic network model for two idealized microbial species meant to illustrate how a minimal metabolism-based mutualistic interaction could occur. The networks of the two individual species are represented in panels **A** (red metabolites) and **B** (blue metabolites). The joint model, in which the two species share the same environment, is depicted in panel **C**, while the specific configuration of active fluxes in the mutualistic regime is shown in panel **D**. The two individual organisms differ only in their internal metabolic reaction (R1), while they have the same metabolites (X, Y and Z), the same uptake/secretion properties, and same usage of precursors to produce biomass. Arrows represent reactions, and encode information about reversibility (single vs. double arrow). The boxes represent distinct metabolic compartments: each organism has an extracellular (EX1, EX2) and a cytosolic (CYT1, CYT2) space. This level of compartmentalization is usually sufficient to appropriately model individual species. In this way, constraints on transporters (reactions TX, TY, TZ) can be decoupled from the constraints on environmental availability of nutrients (reactions EX, EY, EZ). In panels **E** and **F** we display each species as a stoichiometric matrix, in which columns correspond to reactions and rows to metabolites (see Methods for more details). For simplicity, we show here only the nonzero elements of each matrix. In building the joint model (see panels **C**, **G**) the two individual species (maintaining their color code) are embedded in a new environmental compartment (ENV), in which metabolite availability is controlled, as for individual models, through exchange reactions EX, EY, and EZ. The former exchange reactions EX, EY, and EZ of individual species are relabeled in the joint model as shuttle reactions SX, SY, and SZ. Being able to distinguish between shuttle reactions (which allow to artificially control the flow of a metabolite through the boundary of an organism) and transport reactions (TX, TY, TZ, associated with specific enzymes, which could possibly catalyze multiple transport reactions of different molecules) is an important subtlety of our framework, essential for a correct implementation of the SEM algorithm (see Methods). Notice that the stoichiometric matrix for the joint model (panel **G**) is composed of two blocks that are exactly the stoichiometric matrices for the individual species (panels **E**, **F**), with the addition of appropriate stoichiometric coefficients to encode the exchange and shuttle reactions (first three rows and first three columns). If, in the joint model, metabolite X is the only nutrient available in the medium, the two species will be forced to engage in a mutualistic interaction in order to survive (i.e. produce each its own biomass). This is illustrated in panel **D**, where the direction of the arrows reflects active fluxes under this specific condition. The ensuing mutualism is due to the fact that none of the two organisms can produce on its own all three biomass component, and each needs to receive a molecule from its partner.

As a first step in our analysis we asked whether, using stoichiometric models, we could reproduce three metabolic interactions depicted in Figs. 2 and 1. The first, simplest interaction (Fig. 2D) is an elementary case of syntrophy in a toy model. The second interaction (Fig. 1A) is the previously modeled [33] naturally occurring interaction between a hydrogen producing bacterium and a methanogen archaeon [38], [39]. The third (Fig. 1B) is an obligate mutualism between two strains of yeast engineered to be auxotrophic for lysine (Lys-) and adenine (Ade-) respectively [19]. In each case, we built a joint model for the organism pair by combining their stoichiometric matrices into a single ecosystem-level stoichiometry (Fig. 2). This unified stoichiometry involves the creation of a new compartment (the joint environment) that can communicate with the individual species and serves as an interface for the description of environmental nutrient availability (see Fig. 2 and Methods). In the cases of Figs. 2 and 1B, upon building the joint stoichiometry, we could verify that the pairs of organisms could grow only syntrophically. Similarly, for the case of Fig. 1A, we verified that our model implementation reproduced the unidirectional flow of nutrients responsible for the symbiotic relationship between the two species.

**Figure 3:**
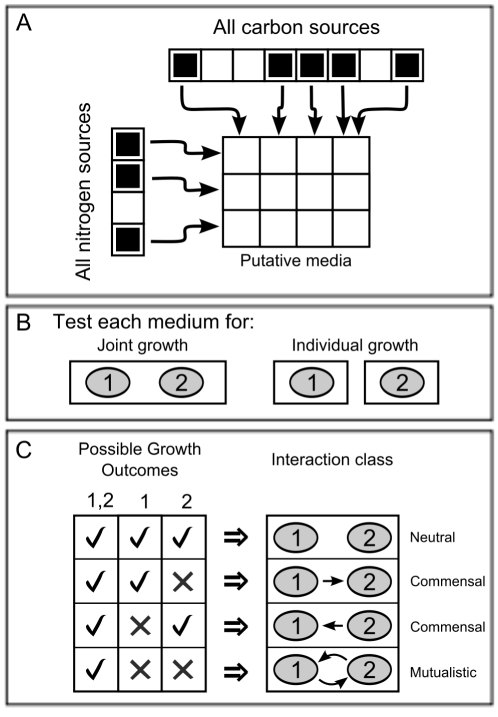
Schematic representation of the pipeline used to search for interaction-inducing media (SIM). The SIM algorithm is capable of identifying a large number of growth media predicted to induce symbiotic interaction between different microbial species. The algorithm is seeded with an initial medium (e.g. containing a specific carbon and a specific nitrogen source) that can sustain growth of a given joint pair of species. Then **A** the algorithm searches for all metabolites (or sets of metabolites) that can substitute the original carbon source, and subsequently all the metabolites that can substitute the original nitrogen source (black squares in corresponding arrays). These selected carbon and nitrogen sources are combined in all possible ways to give rise to a set of putative media that can sustain growth of the joint model (matrix in the figure). In the next step **B** the algorithm loops through each putative medium identified in **A**, and tests for growth of the joint pair as well as of each species individually. Finally **C** the different possible outcomes provide predictions of types of interactions induced by each medium. The actual algorithm, which takes into account more than two elements, is described in detail in the text and Methods.

**Figure 4:**
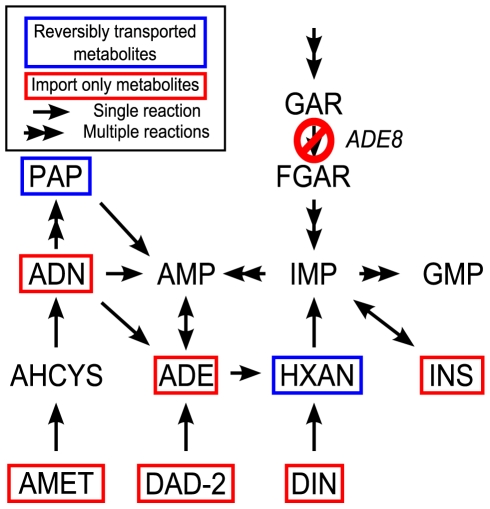
Schematic representation of the metabolic network around adenine in *S. cerevisiae*. This diagram illustrates why only certain metabolites are predicted to be exchanged under the syntrophy-inducing medium used in our computational implementation of the original experimental setup. Only the major metabolic steps around adenine synthesis in yeast are included for simplicity. Nodes represent metabolites, single arrows represent single biochemical reactions, and multiple arrows represent chains of biochemical reactions. Metabolites that can only be imported (but not exported, i.e. unidirectional transporters) according to the stoichiometric model [38] have a red rectangle around them, while metabolites that can be both imported and exported (i.e. have reversible transporters) have a blue rectangle around them. Exchange metabolites are restricted to the set of metabolites the can be imported by the recipient and exported by the provider organism.

We next asked whether the stoichiometric implementation of organism pairs could be used to generate predictions of the metabolites exchanged between the two species upon symbiotic growth. Towards this goal we developed an algorithm that allows us to predict what metabolites need to be exchanged between two species in order to survive under a given environmental condition (Fig. 3). Our search for exchanged metabolites (SEM) algorithm constitutes an extension of flux balance modeling, applied to the unified ecosystem-level stoichiometry (Fig. 2) [31]. SEM is based on a mixed integer linear programming algorithm that identifies the fewest number of metabolites exchanged between individual species, under the constraint that both organisms must still be able to produce biomass at a rate larger than a given minimal threshold. For a particular medium, SEM can recursively find multiple optimal or near-optimal solutions, though it is not guaranteed to identify all possible ones (See Methods for more details). In the toy model (Fig. 2C) and in the methanogenic pair (Fig. 1A) we recovered the expected exchange metabolites. The results were less obvious in the case of complementary yeast auxotrophs (Fig. 1B). In this case, upon applying SEM to the yeast pair, we found two feasible sets of exchanged metabolites under the glucose minimal medium used in the experiment. These sets both use lysine and then either adenosine-(3,5)-bisphosphate (PAP) or hypoxanthine (HXAN) (Fig. S2). While the observed exchange of lysine confirms the intuition, reflecting the deficiency of one of the engineered organisms (Lys-), it was somehow surprising not to observe, for analogous reasons, a predicted exchange of adenine. The reason for this discrepancy can be explained by looking at the relevant metabolic pathways (Fig. 4). Although several metabolites downstream of the *ade8* reaction might restore flux through the rest of the adenine biosynthesis pathway, based on the stoichiometric model [40] only PAP and HXAN can be reversibly transported. As indicated by the metabolic pathway map, both of these metabolites are easily converted into adenosine via mechanisms that circumvent the knocked out enzyme of the Ade- strain. Thus our algorithm produced testable predictions on potentially exchanged metabolites between the two yeast strains in the engineered syntrophic pair.

So far, we have shown that joint stoichiometric models can be helpful in describing known cases of symbiotic interactions, providing novel testable predictions. This analysis, however, has been limited to a single medium composition. Can one generalize this approach, and try to identify a multitude of media that would impose mutualism or commensalism between any two species? To address this question, we developed an algorithm for the search of interaction-inducing media (SIM), aimed at finding a large number of minimal or near-minimal media that are predicted to induce interactions between two given microbial species (see Methods). As a preliminary step for the SIM algorithm, we assemble a list of metabolites that are usable by at least one of the two organisms, based on the stoichiometric models. The algorithm then starts by assigning a single minimal set of metabolites (i.e. a growth medium) that allows both organisms (in their joint pair configuration, as in Fig. 2C,F) to grow with a rate that is above a given threshold (Table S2). This medium is minimal in the sense that removal of any one metabolite makes it impossible for the pair to grow. This minimal medium is also chosen so as to avoid (if possible) nutrients that contribute more than one essential element (e.g. avoiding amino acids, which can serve both as carbon and as nitrogen sources). Next, we perturb this initial medium by removing its carbon source metabolite, causing the modified medium not to sustain growth. We then identify a substitute metabolite (or metabolite set) that restores the capacity for growth (see Methods for a more detailed description). This process is repeated by iteratively removing each possible carbon-contributing metabolite found in the previous step, resulting in a set of feasible carbon sources (black squares in carbon source array in Fig. 3A). This perturbation loop is repeated for different elements (e.g. nitrogen, Fig. 3A). The arrays of feasible nutrients contributing different elements (C and N in the example of Fig. 3; C, N, P, S in the real calculations) are then used to construct a matrix of all possible combinations (Fig. 3A). Each of these combinations constitutes a medium that can putatively sustain growth of the organism pair. The next step is to test whether each of these media indeed sustains growth of the pair, and whether it can sustain growth of each species on its own (Fig. 3B). Even if a medium allows both species to grow, this does not imply that it will necessarily induce mutualistic growth. In fact, some of these media could simply be minimal media that can be used to grow both species individually (see Methods). Other media could be supporting commensal growth, i.e. allow one species to grow, and to produce a metabolite necessary for the other species to survive under the same conditions. Finally, some of the media may sustain growth of both species, without allowing either of the two individual species to grow on its own. Within these media, therefore, the two organisms would be able to survive only by exchanging essential metabolites, in an obligate syntrophic or mutualistic interaction. Computationally, testing for growth of the joint pair and of each individual species, allows us to easily classify the type of interaction induced by each medium (Fig. 3C). The details of the algorithm are described in the Methods section. Note that here we do not take into account the specific cost that an organism would incur to produce a metabolite that can benefit another organism. Hence, we do not distinguish, for example, between commensalism and parasitism (see Table 1).

We first applied SIM to the pairs of organisms presented in Figs. 1A and 1B. For the methanogenic pair of *Desulfovibrio vulgaris* and *Methanococcus maripaludis* (Fig. 1A), SIM identified only six simple media that can sustain growth of the joint organism pair. One of these six media corresponds to an experimentally tested environmental condition, and is the one imposed in the original model. It contains lactate, ammonia and di-hydrogen sulfide. Under this condition, *D. vulgaris* is predicted to utilize lactate and be able to grow on its own, while *M. maripaludis* is not, and can only grow in presence of the H_2_ and formate secreted by *D. vulgaris*. One aspect that the model does not capture explicitly at this point is the benefit that *D. vulgaris* receives from its association with *M. maripaludis*. This benefit is due to the fact that H_2_ consumption by *M. maripaludis* reduces the partial pressure of the gas, allowing *D. vulgaris* to keep producing H_2_ in a thermodynamically advantageous way. In addition to this canonical medium composition, our approach predicts five additional media that allow for growth of both organisms. One of these is predicted to induce another commensal interaction, in which *D. vulgaris* reduces sulfate to sulfide, which is then also shared with *M. maripaludis*. This interaction, however, may not be feasible, as there is some experimental evidence that suggests that sulfate reduction does not occur alongside methane production [14]. Interestingly, the remaining four media are predicted to induce obligate (thermodynamics-independent) syntrophy (Table 2). The mutualistic interactions arise because, according to the model, *M. maripaludis* is capable of fixing nitrogen to ammonium and extracting ammonium from alanine. Ammonium can then be utilized by *D. vulgaris*, which otherwise lacks the capacity to obtain it endogenously. These nitrogen-related interaction predictions are yet to be tested, but previous work has verified that Methanococcus is both able to fix nitrogen [41] and use alanine as a nitrogen source [42]. It is important to emphasize that we are considering here a rather small number of media, which do not include certain metabolites that *D. vulgaris* is known to be able to metabolize, such as pyruvate, ethanol, malate and fumarate [43], [44]. This is a consequence of the fact that the specific stoichiometric models available for these organisms are not genome-scale, but rather encompass only a subset of known metabolism pathways (approximately 100 reactions each).

**Table 2:** Predicted feasible media for the methanogenic community of Fig. 1A.

Key Nutrients Used			Exchange metabolites		Byproducts
Nitrogen	Sulfur	Carbon	*D. vulgaris* to *M. maripaludis*	*M. maripaludis* to *D. vulgaris*	
NH_3_	H_2_S	Lactate	H_2_,Formate		Acetate, CO_2_, CH_4_
NH_3_	SO_4_	Lactate	H_2_,Acetate,H_2_S		Acetate, CO_2_, CH_4_, H_2_S
Ala	H_2_S	Lactate	H_2_,Formate	NH_3_	Acetate, CO_2_, CH_4_
Ala	SO_4_	Lactate	H_2_,Formate,H_2_S	NH_3_	Acetate, CO_2_, CH_4_
N_2_	H_2_S	Lactate	H_2_,Formate, Acetate	NH_3_	Acetate, CO_2_, CH_4_
N_2_	SO_4_	Lactate	H_2_,Formate, Acetate,H_2_S	NH_3_	Acetate, CO_2_, CH_4_

Exchanged and produced metabolites are also indicated. The first medium has been previously experimentally determined, and corresponds to the one used in [33]. The second medium induces a commensal interaction, while the last four media are predicted to give rise to mutualistic interactions.

**Figure 5:**
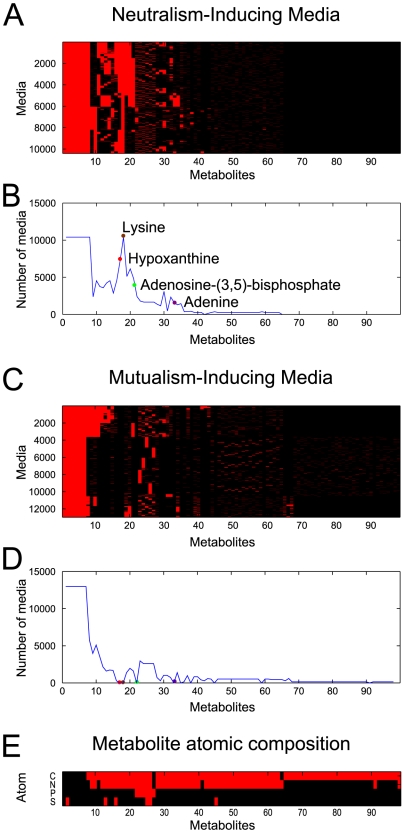
Metabolite usage predicted across interaction-inducing media in the pair of yeast strains from **Fig. 1B**. Metabolites in all panels of this figure are clustered according to their presence in all media analyzed (see Methods). **A** Binary map of metabolite usage (black = not used; red = used) across all neutralism-inducing media. This emphasizes that some metabolites are used in all media, while others are never used. A large set of metabolites is used in different combination, forming a complex map of mutual capacities to compensate each other. **B** By looking at the overall frequency of metabolite usage across all neutralism-inducing media, one can see that lysine and adenine, or their derivatives, are always present, as expected. **C** The binary map (as in panel **A**) for all mutualism-inducing media. **D** Frequency of metabolites across all mutualism-inducing media. These media never contain the lysine and adenine derivatives observed above. Metabolites appearing at high frequency (positions between 20 and 30 on the x axis) correspond to carbon sources swapped between the two strains. **E** The atomic composition of metabolites from the above maps. This chart allows one to track the distribution and overlap of different atomic sources across metabolites.

In the case of the engineered yeast pair (Fig. 1B), SIM led to the prediction of a total of 36212 distinct media that allow for growth of both yeast strains in the joint model (Fig. S1). Of these, a total of 12981 media (35.8%) do not support growth of the individual strains (mutualism-inducing media), 12817 media (35.4%) can sustain growth of one of the organisms but not the other (commensalism-inducing), and 10414 (28.8%) can sustain growth of each strain individually (neutralism case). The computation of these media for the synthetic yeast pair offered us the opportunity to obtain more insight into how the algorithm performs, and into the biochemical rationale for patterns of interactions observed. Specifically, we compiled a metabolite-by-condition usage matrix ***M*** whose element *M_ij_* is equal to one if metabolite *j* is used in condition *i*, and zero otherwise, as predicted by SIM (Fig. 5 and Fig. S3). By clustering the columns (i.e. metabolites, see Methods for details) of the ***M*** matrix, it is apparent that some metabolites are required under all conditions, while other metabolites are not essential, being only required occasionally, often serving as alternatives to nearby metabolites (Fig. 5). Furthermore, one can separate the ***M*** matrix into sub-matrices pertaining to the four different classes of interactions (neutralism, mutualism, and the two commensal), and detect specific patterns of interaction that can be reconciled, e.g., with the biochemistry of the syntrophic pair (Fig. 5 and Fig. S3). One may also ask whether it is possible to discriminate between different types of interactions by performing an unsupervised clustering of the different media. Upon implementing a k-means clustering of neutral and mutualistic media, we found that the two sets of media do partition significantly more than random (p-val <10^−300^, see Methods), but in a way that is too weak to allow for a straightforward classification.

The interaction class specificity of certain metabolites is best highlighted in Figs. 5B, D, where, for each metabolite, we enumerate the total number of media per interaction class. This provides a comprehensive picture that complements the analysis of exchanged metabolites described above. In general, this type of representation may be useful in trying to design or prioritize media for experimental testing and applications, where one may want to give top preference to media that are mostly found in one class of interactions (e.g. syntrophic), and that are common to a large number of identified media (see Text S1). Different, biologically more relevant criteria for prioritizing metabolites (and consequently media that contain them) may be envisaged, for example based on the number of reactions each metabolite participates in (see Methods, Table S4).

**Figure 6:**
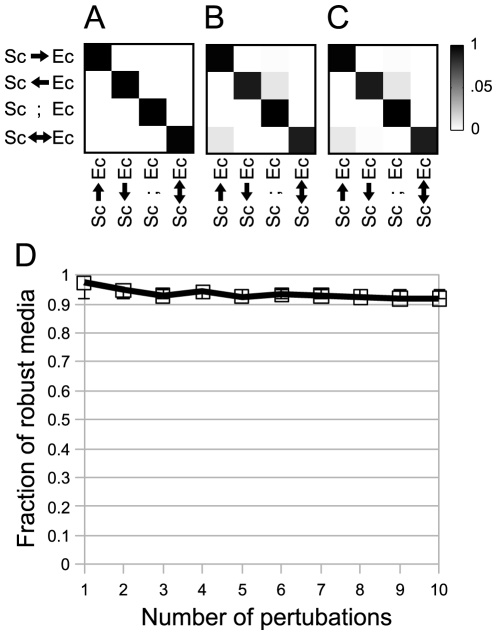
Robustness analysis of the joint *E. coli* – *S. cerevisiae* model. We evaluate the robustness of our approach by estimating the chance that a predicted interaction (e.g. mutualism) will change (e.g. to commensalism) when an organism's metabolic network is perturbed. In A, B and C this chance is displayed as a probability transition matrix, where darker shades indicate larger probability. Each row and column corresponds a different type of interaction (Sc = *S. cervisiae*; Ec = *E. coli*). Here the perturbations (100,000 for each matrix) are **A** individual reaction deletions, **B** reaction additions, and **C** combined instances of one addition and one deletion. The cumulative effect of multiple random addition or deletions (from one up to 10, corresponding to ∼0.25% of the organisms' reaction set) is illustrated in panel **D**. This graph shows the probability of not changing interaction class as a function of the number of perturbations implemented. This analysis shows that interaction classes are quite robust even when faced with multiple insults.

The detailed analysis of the three test cases described so far indicates that the SEM and SIM algorithms are helpful in identifying true interactions and providing insight into the biochemical pathways underlying experimentally observed or putative interactions. As a first step towards novel synthetic ecology predictions, we studied the spectrum of possible interactions between *E. coli* and *S. cerevisiae*. This is motivated by the fact that the individual stoichiometric models for these two organisms are possibly the best curated and thoroughly tested experimentally [30], [45]–[47]. The *E. coli* - *S. cerevisiae* pair could be seen as a reference system for future experimental testing, as well as a good benchmark for performing sensitivity analyses of our algorithms. By applying the SIM algorithm to this pair we identified ∼11.6 million media (Fig. S6), out of which 4.7% are mutualism-inducing, 3.3% and 75.3% are commensalism-inducing (*S. cerevisiae - E. coli* and *E. coli* – *S. cerevisiae* respectively), and 16.8% are neutralism-inducing. In Table S4 we analyze in detail two solutions selected based on the prioritization criteria described above. Fig. 6 illustrates the results of multiple types of sensitivity analysis performed on the *E. coli* – *S. cerevisiae* pair. In particular, it can be seen that the interaction class predictions are highly robust with respect to individual perturbations that remove (Fig. 6A), add (Fig. 6B) or simultaneously add and remove (Fig. 6C) individual reactions from the stoichiometric models. Furthermore, robustness does not decrease significantly, on average, for at least ten cumulative gene addition/removal perturbations (Fig. 6D).

It is in principle possible to extend our computation of interactions to any organism for which a stoichiometric reconstruction is available. Here we present a computation of all possible pair-wise interactions between seven bacteria of relevance to health (first three) or environmental (last four) applications, namely *Escherichia coli*, *Helicobacter pylori*, *Salmonella typhimurium*, *Bacillus subtilis*, *Shewanella oneidensis*, *Methylobacterium extorquens*, *and Methanosarcina barkeri*. These specific organisms were chosen based on a balance between the following criteria: (i) they span wide spectra of function, environment and taxonomy; (ii) most of them have well characterized laboratory strains which could be used for future experimental testing; and (iii) they have publicly available stoichiometric models. While all stoichiometric models used here have undergone manual curation and some form of experimental validation, it is important to keep in mind that different models may have different levels of agreement with experimental observations. Thus, predicted interactions between arbitrary pairs should be evaluated in light of the expected fidelity of the corresponding individual stoichiometric models.

**Figure 7:**
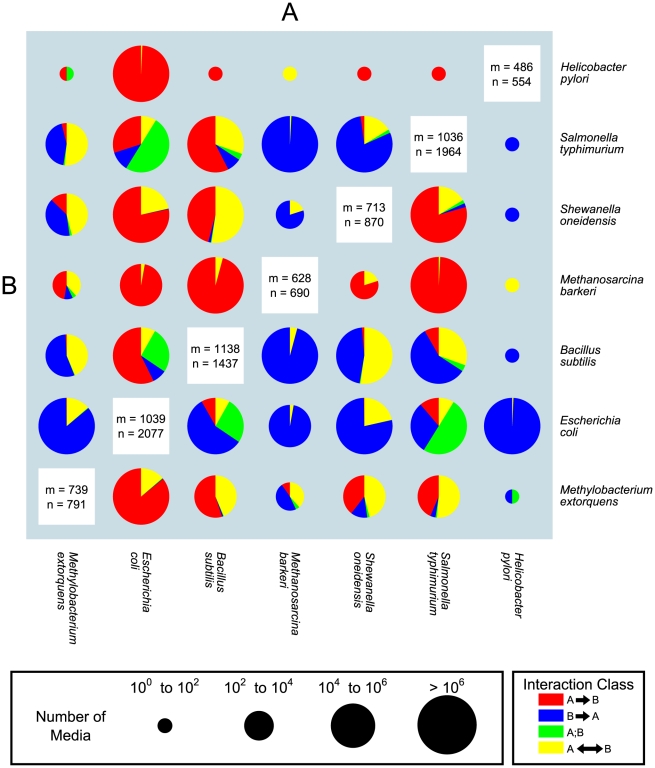
Predicted map of pairwise interactions between seven microbial species. The size of the pie chart is representative of the number of media that allow growth of both organisms, as defined in the pie chart size legend to the right. The relative amount of each interaction type is represented by the proportion of each wedge of the pie. Details on the numbers and types of interactions for each pair of organisms can be found in Table S1. The values of *n* and *m* reported in the white boxes on the diagonal indicate respectively the number of reactions and metabolites present in each stoichiometric model.

The results of this interaction analysis are summarized in the matrix of pie charts found in Fig. 7 (see also Table S1). The number of interaction-inducing media for a pair of organisms (size of each pie chart), as well as the proportions of different types of interactions, can vary significantly between pairs, from more than three hundred million for the *E.coli* - *S. typhimurium* pair, to only one in the *H. pylori* - *M. barkeri* case. At a first glance, one can identify several trends in the patterns of predicted inter-species interactions. For example, *E. coli* is predicted to be able to interact with most organisms in a large number of different environments. These interactions appear to be dominated by commensalism, where *E. coli* acts as the provider. This may be due to a combination of *E. coli*'s ability to survive on a variety of carbon sources and of its capacity to export a number of byproducts. In contrast, *H. pylori*, except for the interaction with *E. coli*, has very few interaction-inducing media and frequently acts as a recipient, possibly reflecting its near obligatory parasitic nature [48]. Some organisms lie between these two extremes, e.g., the archaeon *M. barkeri*, which is predicted to interact with other species in a moderate number of environments, most of which induce commensal interactions that have *M. barkeri* a*s* the recipient. In general, pairs of organisms appear to be dominated only by a few interaction classes. In addition, specific organisms tend to have an overall specific role in interactions with all species. For example, *E. coli* and *B. subtilis* are largely on the giving end of commensal interactions, while *M. barkeri* and *H. pylori* are mainly on the receiving end, and a sizable portion of *M. extorquens* and *S. oneidensis* interactions are mutualistic. Furthermore, some pairs display a large proportion of neutral interactions, i.e. can individually grow on a lot of common minimal media. This is most prominent for the *E. coli* and *S. typhimurium* pair, for which such an outcome may be expected, based on the fact that they tend to occupy a similar environmental niche. What might be less intuitive is that, for this same pair, we found also numerous environments that induce commensalism and mutualism.

For each pair of organisms it is possible to analyze in detail the environments identified, and use the SEM algorithm to determine what nutrients might be exchanged between the two organisms. For example, a mutualistic interaction between the bacterium *E. coli* and the archaeon *M. barkeri* is explored in detail in Fig. S5. This interaction is particularly interesting as a similar bacterium/archaeon pair may have been implicated in the rise of the primordial eukaryotic cell. For certain combinations of organism pair and growth media the SEM algorithm becomes computationally very heavy, and impractical. An alternative heuristic for identifying possible minimal sets of exchanged metabolites can be implemented in these cases (see Methods). We applied this alternative method to identify the exchanged metabolites in two cases of mutualism between *S. cerevisiae* and *E. coli* and two cases for the *E. coli* - *H. pylori* pair (Table S4). The relatively simple set of exchanged metabolites of the *S. cerevisiae* - *E. coli* pair provides novel experimentally testable biological hypotheses. The more complex metabolic exchange in the *E. coli* - *H. pylori* pair may reflect the parasitic nature of *H. pylori*.

**Figure 8:**
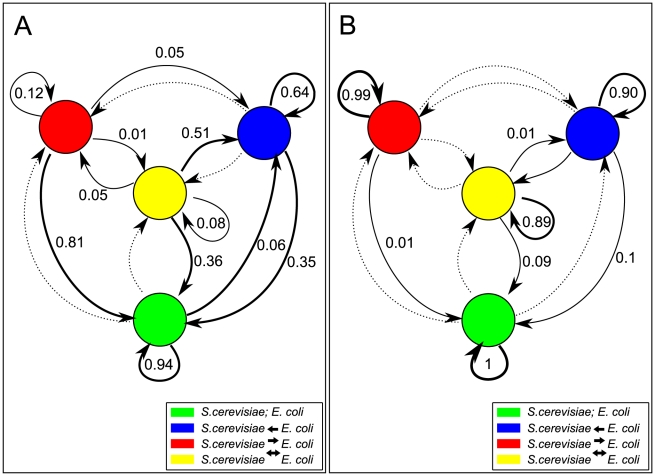
Graph of transition probabilities between different types of interactions in the joint *S. cerevisia*e - *E. coli* model upon environmental and reaction perturbations. These graphs show the chance of transition from one interaction type to another (as in Fig. 6) computed for either **A** environmental or **B** reaction network perturbations. For reaction network perturbations, the transition chances are the same ones computed for Fig. 6C. For environmental transitions, we performed perturbations to the growth media by swapping a randomly chosen environmental metabolite with a different random one. Arrows thickness and corresponding number indicate the probability of each interaction class (neutralism, mutualism, and two commensalism classes) remaining unchanged (self-arrows) or transitioning to a different interaction class. Dashed arrow represents transition probabilities of less than 0.01. See methods and text for more details.

Interestingly, almost all pairs in Fig. 7 appear to be potentially capable of engaging in mutualistic interactions (yellow portion of pie charts), given the appropriate growth medium. This is in sharp contrast with the relative paucity of neutralism-inducing conditions (Fig. 7, green portion of the pie charts). In general, the fact that neutralism-inducing interactions are rarely observed means that, upon exploring the space of next-to-minimal media that sustain a pair of organism, it is much more difficult to find media that sustain each organism on its own than it is to find media that support lifestyles involving unidirectional or bidirectional exchange. To some extent this may be expected, given that our algorithm searches for parsimonious solutions which guarantee growth of a pair of species. However, since it was not obvious *a priori* whether symbiotic interactions were at all possible, one may take these results as an indication that nutrient-poor environmental conditions are expected to be dominated by symbiotic interactions. Such a view point would offer the opportunity to use our approach as a quantitative modeling framework for partially understanding and estimating microbe unculturability in the wild. Moreover, it is possible to envisage simple simulations of the long-term dynamics of symbiosis based on estimates of the probabilities of transitions between different types of interactions upon environmental fluctuations. To exemplify this idea, we computed a matrix of transition probabilities for the *E. coli* – *S. cerevisiae* pair (Fig. 8A) (see Methods). A striking feature of this graph is the high transition probability between different states, suggesting a major and dynamical role of environmental fluctuations in determining microbial community lifestyles. In this case, the mutualistic state is quite unstable, possibly a consequence of the distinct environmental niches in which the two organisms belong. The fluidity of symbiosis under environmental perturbations is especially interesting in comparison with the corresponding graph for genetic perturbations, displaying a high degree of robustness, i.e. stability of individual states (Fig. 8B, extension of the data from Fig. 6).

## Discussion

Understanding natural and engineered microbial ecosystems is an ongoing challenge, relevant to multiple disciplines and applications. It is a challenge that undoubtedly requires a large computational effort, and novel algorithmic approaches. Our proposed method for identifying environments that induce symbiotic interactions is based on genome-scale stoichiometric models of metabolic networks, and can be in principle scaled up to computing interactions between any pair of organisms for which individual genome-scale constraint based models are available [49], [50]. In the current search for symbiosis-inducing environments we varied only metabolites containing carbon, nitrogen, phosphorus, and sulfur. Future extensions may explore larger chemical spaces, which could help identify interactions that are based on the exchange of other essential elements, including metal cofactors such as iron and magnesium. Furthermore, future models could be made more realistic by taking into account the role of fitness cost in determining the evolutionary advantage of different metabolite-sharing strategies.

While our implementation of the SIM algorithm to identify symbiosis-inducing media is currently limited to pairs of organisms, it can be easily extended to predicting media that induce symbiosis between triplets of species, or larger combinations. This follows from the fact that the framework defined in Fig. 2 can accommodate any number of different species, and that the algorithm would only need to check a longer list of options (e.g. all possible three-way interactions) relative to what presented in Fig. 3C. Extending the SEM algorithm for searching minimal sets of exchanged metabolites to three or more organism, however, would be more challenging, and one may need to further develop heuristics such as the one we used for the data in Table S4. In going from pairs to more complex communities of organisms known to populate a given environment, our algorithms would provide putative sets of interaction networks viable for such ecosystem. As of now the computation of the media for a given pair of microbial species takes on the order of 10 CPU hours per 10,000 media tested. This implies that systematic calculations for pairs or triplets between tens or hundreds of species will require high performance computing platforms and optimized algorithms. Given the current high pace of developments in these research areas, we envisage that this will be possible in the near future.

While increasingly detailed genome-scale stoichiometric models are being built and validated experimentally for several species [49], [50], one should keep in mind that these models constitute only coarse approximations of real biochemical complexity, which lack several layers, such as regulatory feedback and many thermodynamic constraints, and are often limited by our knowledge of gene function. Missing pathways, or wrongly annotated ones, as well as different levels of knowledge available for different organisms could bias our predictions, and could give rise to false positive or negative predictions of interactions. As seen in the engineered yeast strain test (Fig. 4, and Fig. S2), the direction of metabolite transport can play a role in defining possible exchanges between species, as can the number and specificity of annotated transporters and listing of non-enzymatic metabolite diffusion capabilities. However, examination of the relative number of transport reactions and their direction across all the species used in this study does not show any major bias (data not shown), suggesting that the different patterns of interactions observed in different organism pairs are not merely a consequence of major discrepancies in level of detail for different species.

In addition to transporters, any individual reaction within an organism's network could have an impact on possible interactions. Hence it would be important to know how sensitive our overall classification of media is to potential annotation errors in the models. We addressed this point by performing multiple types of sensitivity analyses on two different pairs of species (Fig. 6 and Fig. S4). The results of this analysis indicate that our interaction classification is largely robust to missing reactions in the model, suggesting that the patterns depicted in Fig. 7 roughly reflect true biological expectations. Even though our predictions are computationally robust, the ultimate way to determine the predictive capacity of our approach will be experimental testing. Similar to the cycles of refinement for individual genome scale stoichiometric models [49]–[51], we expect that the results from biological experimentation can feed back into refinement of models and algorithms for predicting interactions. For example, if a medium predicted to induce a mutualistic interaction is found experimentally to induce a commensal relationship instead, this would provide useful knowledge about metabolite uptake properties not accounted for in the model (Table S3).

Despite these potential limitations, our community models were successfully used to identify media that allow for mutualistic growth and identify metabolites that are being exchanged in three test cases. Running these processes on the toy system showed us that these algorithms give results that match our intuition and can be manually validated. By applying the algorithm to more complicated scenarios, such as an experimentally proven syntrophic yeast pair, we could recapitulate biological observations and predict additional experimentally testable environments under which similar mutualistic communities should arise.

Bridging across levels of description, one can draw an analogy between epistatic interactions in genetic networks and symbiotic interactions between microbes. The molecular composition of a sterile medium could be seen as an ecological unperturbed phenotype. It could be the case that a given microbial species is unable to grow on such medium. Hence, “perturbing” the environment by inoculating this microbe would give no observable change in the medium composition, i.e. no phenotype. This could happen equally for a second microbial species. However, if the two species are able to grow syntrophically in this environment, inoculation of both microbes (i.e. a double perturbation) will produce a major change in the medium, i.e. consumption of resources used for growth, and generation of novel byproducts. Thus, the combined effect of the microbes is highly synergistic relative to what is expected by observing the two microbes alone. This phenomenon is formally analogous to an extreme epistatic interaction between gene deletions, such as synthetic lethality [45]. Given the increasing amount of data and mathematical expertise generated in the study of genetic interaction networks, we envisage that valuable cross-fertilization will be possible between the field of synthetic ecology and the study of genetic networks.

Our results offer new insight on some evolutionary aspects of microbial ecosystems. Cryptic metabolic interactions may be the source of the unculturablity of many organisms. Since next-generation sequencing technologies can now produce the sequenced genomes of organisms we can not culture, it is possible to construct the metabolic network model for a species that has never been grown in a lab as pure culture. From this model one could infer what metabolites such organism would require from an interaction partner, paving the way for novel experimental testing. The mechanisms responsible for the evolutionary emergence of mutualistic interactions are an unresolved puzzle, though there is evidence that gene loss or horizontal gene transfer may drive some of these processes [52], [53]. Our results suggest an alternative mechanism, driven by environmental changes. Two organisms, initially growing independently of each other in a given environment, may be forced to become a commensal pair upon environmental depletion of a metabolite required by one of the species and producible by the other. A bidirectional co-dependency could ensue from a subsequent environmental change forcing a similar interaction in the opposite direction. Based on our prediction of nutrient sets that support symbiotic interactions, it may be possible to estimate the chances that a random walk in the space of environments will hit a mutualism-inducing or a commensalism-inducing one.

Another aspect that should be stressed is that many of the predicted symbiosis-inducing media may be theoretically feasible, but still not practically viable in a straightforward way, e.g. because the relevant metabolic pathways or necessary transporters may be down-regulated, allosterically inhibited or kinetically unfavorable. Such limitations, as well as potential limitations in metabolite transportability, might be overcome by implementing targeted (e.g. regulatory) mutations, or rounds of experimental evolution [52], [54], [55].

Finally, we envision that our proposed approach of a computationally driven synthetic ecology based on re-designing environments rather than organisms could have several applications. First of all, in analogy with synthetic biology, and as explored already with some artificial synthrophic species, the payback will be partially in terms of understanding interactions by building them. This may be seen as a first step towards building a stoichiometry-based microbial interactome, to help in the interpretation of metagenomic sequencing and microbial ecosystem data. Moreover, in terms of metabolic engineering, using the enzymatic potential of multiple interacting species can greatly expand the space of process optimization possibilities. Generating novel pathways by inducing interactions between different organisms rather than (or in addition to) genetically engineering the genomes of individual species has several benefits. First, one could use the metabolic potential of organisms that may be hard to genetically manipulate. Second, communities may be inherently more stable than individual modified species, in which specific mutations could potentially revert. We anticipate that methods like the ones we propose will be important in developing and analyzing synthetic communities of organisms. Our algorithms can be extended to simulate communities containing more than two organisms, predict gene knockouts that would give rise to mutualistic interactions and eventually entire consortia of microorganisms. Furthermore, the algorithms and methods we developed could be extended to study human health related problems. In addition to understanding interactions in the human microbiome, similar approaches could be used to ask how different cell types interact within a specific tissue and how a pathogen interacts metabolically with the host it infects.

## Materials and Methods

### Definitions and basic formulation of stoichiometric models

Our algorithms use the framework of stoichiometric constraint-based models of metabolic networks, which have been described in detail elsewhere [56], [24], [57]. A stoichiometric matrix (***S***) is used to encode all the information about the topology and mass balance in a metabolic network, including the complete set of enzymatic and transport reactions in the system. Transport reactions, inferred from genome annotations, specialized prediction tools or literature curation, include both protein-catalyzed transport, e.g. ATP-driven transport, or ion-coupled symport/antiport, as well as free diffusion of small molecules (e.g. O_2_, CO_2_, etc.) through the cell boundaries. Element *S_ij_* represents the number of molecules of metabolite *i* participating in reaction *j* (with *i* = 1,…,*m*, and *j* = 1,…,*n*). The stoichiometric matrix ***S*** can be used as the starting point for efficiently generating predictions of metabolic rates (fluxes, *v_j_*, with units mM·(g dry mass)^−1^·(hr)^−1^) at a genome scale, e.g. using flux balance analysis (FBA) [56], [24]. FBA is generally based on two main simplifying assumptions. The first is a steady state assumption, which in matrix form can be expressed as ***S*** *v* = 0. This assumption generates a large number of equality constraints that define the space of feasible metabolic states for the system. Further constraints (e.g. associated with reaction irreversibility of individual reactions) are imposed through inequalities of the type 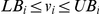, where *LB_i_* and *UB_i_* constitute vectors of lower and upper bounds of reaction *i* respectively. These constraints will be later written concisely as 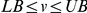. The second step of FBA is an optimization step, in which Linear Programming (LP) can be used to determine feasible flux distributions for some presumed cellular objective (c), subject to the previously described constraints. Typical objectives include maximization of biomass or ATP production, though different optimization approaches are used throughout this work, as illustrated below. Our implementation of flux balance models uses the GNU Linear Programming Kit (GLPK, http://www.gnu.org/software/glpk/) called from a Matlab shell through GLPKmex (http://glpkmex.sourceforge.net/).

### Toy model

The toy model is composed of two simple organism models (Fig. 2, and Fig. S1). Each sub-model contains transporters for three metabolites (X, Y and Z), one biochemical reaction (X→Y or X→Z) and one growth reaction (X+Y+Z→Biomass). Individually organism 1 (red in Fig. 2) can grow on metabolites X and Z, and produce metabolite Y. Organism 2 (blue in Fig. 2) can grow on metabolites X and Y, and produce metabolite Z. If organisms 1 and 2 where grown as a co-culture (i.e. sharing the same environment) they would only need metabolite X to be both able to grow.

### Methanogenic pair model

We implemented a stoichiometric model for the methanogenic syntrophic pair constituted by *D. vulgaris* and *M. maripaludis*, as described in [33], and originally implemented on the FluxAnalyzer platform [58]. The original model was shown to produce valuable quantitative predictions about metabolic interactions between the two species. During growth on lactate, *D. vulgaris* produces H_2_, acetate and formate, all byproducts which *M. maripaludis* can utilize to grow and produce methane. Stolyar *et al.* [33] created biochemical models for each organism and joined them through an intermediate extracellular space. In transferring the model to our simulation platform we performed slight updates to the stoichiometric matrices, as detailed in the supplementary data file on our website, http://synthetic-ecology.bu.edu/. Our flux balance models involve 108 reactions for *D. vulgaris* and 103 for *M. maripaludis*. It is important to mention that these specific stoichiometric reconstructions are not genome-scale (as opposed to the ones used subsequently in our work), and only account for carbon, nitrogen, sulfur and hydrogen atoms.

### Engineered *S. cerevisiae* pair model

To create the joint yeast model, we began with two copies of the newest yeast genome scale metabolic reconstruction iMM904 [40], and modified them to match the biological strains used in [19]. In one model, we identified and disabled the reactions associated with the gene *Lys2*, by setting the upper and lower flux bounds to zero. This modeled strain corresponds to the experimentally constructed Lys- strain, and is unable to grow on glucose minimal media without lysine as a supplement. Similarly, to model the Ade- biological strain we identified and disabled the reactions associated with *Ade8* gene. This resulted in a model that required an adenine supplement to grow on a minimal medium. The strains in [19] had additional mutations that disabled the allosteric regulation of lysine and adenine pathways. As regulation is not represented in our constraint-based models, this aspect was left out. Unless otherwise noted, in the simulated minimal media we limit the amounts of glucose and O_2_, and allow free use of ammonia, sulfate, phosphate and salts.

### Microbial models used in the interaction matrix

The genome scale metabolic models for 5 of the organisms used have been published and are publicly available (*Escherichia coli* [59], *Bacillus subtilis* [60], *Helicobacter pylori* [61], *Salmonella typhimurium* [62], *Methanosarcina barkeri* [63]). For *Shewanella oneidensis* we used an early version of the recently published model [64], provided to us by Jennifer Reed. For *Methylobacterium extorquens* we used a genome scale extension of a previous reconstruction [65], provided to us by Steven Van Dien. Models were imported into Matlab from the XML files using the Cobra toolbox [25]. The metabolites of each model were manually checked for consistency across models.

### Multi-species stoichiometric models

Our approach for generating multi-species model extends the multi-species model employed by Stolyar *et al.*, by introducing a fictitious compartment that represents the extracellular environment shared by both species, in addition to the original extracellular spaces for individual models. Our formulation, for two species 1 and 2 (assumed for this explanation not to have a periplasm) uses the following compartments (represented in square brackets, as in the standard notation used in [59], [40], [66], [67]): [CYT1] = cytoplasm for species 1, [EX1] = extracellular space of species 1, [CYT2] = cytoplasm of species 2, [EX2] = extracellular space of species 2, [ENV] = environment shared by species 1 and 2. For a metabolite X_i_ that can be exchanged between the environment and a given species (say species 1), we define the following reactions (where Y and Z are potential cofactors involved in transport across the cell membrane):

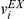
 = Exchange flux for X_i_ X_i_ [ENV] < = >

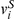
 = Shuttle reaction for X_i_ X_i_ [ENV] < = > X_i_ [EX1]

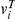
 = Transport for X_i_ X_i_ [EX1] + Y < = > X_i_ [CYT1] + Z

In previous formulations of joint models for different species or organelles [31]–[33] the extracellular spaces for the two interacting organisms (i.e. [EX1] and [EX2]) was collapsed into a single compartment, with no need for [ENV]). Here, by introducing an extra layer (and the extra shuttle reactions) we make it much easier to monitor what metabolites are being transported through the membrane. For example, if metabolite X_i_ is transported in and out of the cell through several different transporters, in order to know whether there is a net influx or efflux of X_i_ we would have to add up all the transporter fluxes. In our formulation, this is achieved simply by looking at the shuttle reaction flux. In a single species model, this would have been easily achieved by observing the exchange flux, but the extra degrees of freedom entailed by the multi-species model requires this extra layer of description. In addition, this formulation makes it much easier to implement our search algorithm for exchanged metabolites (SEM), in which we want to minimize the number of exchanged metabolites irrespective of the number of transporters available for each metabolite. The other important aspect of this distinction between exchange and shuttle fluxes is that, in terms of constraints, we have independent control on what molecules are environmentally available versus what molecules we want to make available for individual species. While this feature has not been used in the current work, it may be useful in future developments. The proposed formulation could serve as a standard way of building ecosystem-level stoichiometric models.

### Search for Exchanged Metabolites (SEM) algorithm

We have developed a mixed integer linear programming algorithm to identify a minimal set of possible exchanged metabolites between two organisms 1 and 2 that can grow simultaneously under a specified condition. Solving this problem requires imposing additional constraints to the regular mass balance and capacity constraints. First, since we require growth of both organisms, we fix the minimal growth rate of both organisms (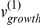 and 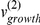) to an arbitrary minimal amount (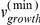 = 0.1 in our simulations):
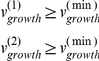
A second constraint derives from the need to identify the set of metabolites that can be exchanged between the two species. This can be done by finding the intersection *TM^(1 and 2)^* between all the metabolites that are potentially transportable in the first and in the second model (metabolite sets *TM^(1)^* and *TM^(2)^* respectively). Each interchangeable metabolite *i* in *TM^(1 and 2)^* is associated with two shuttle reactions 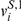 and 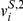 importing the metabolite into individual species, and one exchange reaction 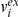, mediating its transport to the common environment. The condition of mutual exchange of metabolite *i* can then be expressed as the following constraint:
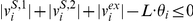
where *L* is a large number (“infinite”), and *θ_i_* is a binary variable which assumes a value of 1 if metabolite *i* is transported between the two species, and 0 otherwise.

Identifying a minimal set of exchanged metabolites amounts then to minimizing the sum of the *θ_i_* variables over all metabolites. Overall, the optimization problem can be expressed as follows:
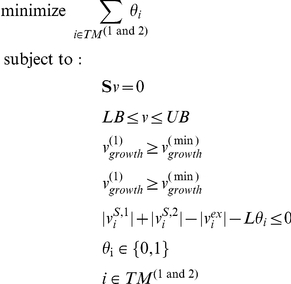
In most cases, the joint flux balance model for two interacting species in a given medium can have multiple feasible flux solutions. Correspondingly, under a given growth condition there may be multiple equivalently minimal sets of exchanged metabolites. To address this degeneracy, we developed an algorithm that systematically identifies a large number of exchange metabolite sets. We reasoned that a set of exchanged metabolites will likely be a minimal set that allows for growth of both organisms. Hence, if we remove any one metabolite from the exchanged set, growth of both organisms is not possible without adding in at least one other metabolite into the set. Applying this algorithm in an iterative way allows us to identify multiple alternate exchange sets. At any given step, one metabolite is removed from the last solution, forcing the solver to find a substitute exchanged metabolite at the next iteration. This process is repeated until no more feasible solutions can be found.

### Alternative exchanged metabolite identification method

For very large pairs of stoichiometric models, the SEM algorithm described above may be impractical. Therefore, we have devised a heuristic 2-steps alternative method that is more easily scalable to large systems. In the first step of this approach we solve a modified FBA problem for the joint pair of organisms using Linear Programming. Specifically, we minimize the sum of shuttle reactions fluxes that do not involve metabolites found in the current medium. This means that the search space is defined by
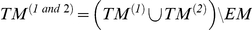
where EM is the set of metabolites contained in the current growth medium. All constraints are the same as described for the SEM algorithm. As opposed to SEM, in this optimization problem we minimize the sum of the absolute values of the fluxes, hence removing any non productive (e.g. cycles) exchange of metabolites:
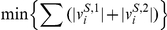
This first step does not necessarily find a minimal set of metabolites that mediate the interactions, but rather one of many possible feasible set. As a second step, we can then apply the SEM algorithm where we limit TM to the metabolites found in the first step. The final set identified will be minimal (in the sense that removal of any metabolite will lead to infeasibility), but may not have a globally minimal count of exchanged metabolites, due to the intermediate step before SEM.

### Search for Interaction-Inducing Media (SIM) algorithm

Here we describe the heuristic for identifying the set of media that support growth of multi-species co-cultures, and predicting the class of interaction they induce (see Fig. 3 for more details). After building a joint stoichiometric model as previously defined (Fig. 2), we identify an initial minimal medium (MM) that allows for positive growth rate of both organisms. In this work, we choose this initial medium manually, so as to select nutrients that are common to most organisms, and that constitute single element sources (e.g. do not contain both C and N). Our MM contained, for all pairs, succinate, ammonium, inorganic phosphate and sulfate, as well as oxygen and minerals. Then, in individual pairs, we included a minimal number of additional secondary metabolites (e.g., co-factors) as needed (see Table S2).

The core of the SIM algorithm is a function that identifies all possible metabolites (or sets of metabolites) that can substitute in the medium an initially available source for a given atom. In the current work we focus on identifying different sources for carbon, nitrogen, sulfur, and phosphate only. The analysis could be in principle extended to other atomic contributions, including cofactor metals, such as iron. The core function in SIM is recursive, and is best described, for a specific atom A, through the following pseudo-code (where CM is the Current Medium being evaluated) (see also Fig. 3):

<Initialize CM = MM

Initialize PM = {All environmental metabolites}\CM

Function Find_Replacement(A, CM, PM) {

Temporary Medium (TM) = CM\{molecules that contain A}

Replacement Sets (RS) = empty set of replacement metabolites.

X = Find X∈PM such that V_growth_>0 on union(TM, {X}) (SMM algorithm, see below)

if ( X is not empty ) {

TM = union(TM, {X})

Remove X from PM

RS = Find_Replacement(A, TM, PM)

RS = [[X], RS ];

}

return RS

}

Note that the real algorithm (see Matlab scripts at http://synthetic-ecology.bu.edu/) takes into account the fact that it may not be possible for a single metabolite to substitute a previous one. In such case the function will continue searching for an additional metabolite that would allow nonzero growth. Hence, it may be the case that at a particular iteration, two molecules are compensating for the initially removed one. In the subsequent step, the algorithm will bifurcate, and try to remove each of these molecules individually.

In the next step, we generate a large set of possible media by determining all possible combinations of replacement metabolites for different atoms (Fig. 3A). We then check each predicted medium for growth of the joint and two individual models by applying FBA (Fig. 3B). Those media that allow for growth of the two organisms in the joint model, but not the individual models induce mutualistic growth through the exchange of metabolites. Media that sustain growth of only one individual organism, in addition to the pair, induce commensal interactions. Finally, those media that sustain growth of both individual organisms constitute cases of a neutral interaction (Fig. 3C, Table 1).

Here, we implement SIM based on a single initial MM. However, the specific choice of MM may influence the composition of the media predicted by SIM. To address this question, we implemented a sensitivity analysis, by focusing on a specific pair of organisms (yeast syntrophic pair), and recalculating the interaction-inducing media based on different choices of the initial carbon sources (glucose, pyruvate, acetate, fructose). Regardless of which initial medium was used, the same metabolites were identified as being viable carbon sources in the different trials. The identified media (i.e. combinations of the above metabolites) were almost identical (average 97.4% overlap±2%), regardless of the initial medium used.

### Search for Minimal Media (SMM) Algorithm

A minimal medium is defined here as a set of metabolites that allows for a feasible solution with positive growth rate, and such that removal of any metabolite from the set would force the system to have no solution, or solutions with zero growth. To find the metabolites that belong to a minimal media, we implemented a mixed integer linear programing algorithm similar to what has been previously used in [68], [69]. As a first step we identify the set {*v^EX^*} of exchange reactions (labeled as (1) in the section *Multi-species stoichiometric models* above). We then solve a minimization problem which uses, in addition to the usual FBA constraints: (i) a constraint on minimal growth rates, as described for SEM (
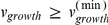
) and (ii) a constraint expressing whether or not metabolite *i* is utilized (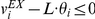). Here, the binary variable *θ_i_* assumes a value of 1 if metabolite *i* is transported between the two species, and 0 otherwise.

Identifying a minimal set of metabolites in a medium then amounts to minimizing the sum of the *θ_i_* variables over all metabolites in {*v^EX^*}. Overall, the optimization problem can be expressed as follows:
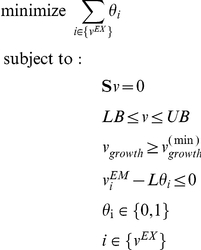



### Clustering of metabolites and media found with SIM

To cluster the metabolites of all the media identified in the syntrophic yeast pair (Fig. 5 and Fig. S3), we compiled a metabolite-by-condition usage matrix ***M*** whose element *M_ij_* is equal to one if metabolite *j* is used in condition *i*, and zero otherwise. We clustered the columns (i.e. metabolites) of the ***M*** matrix, by implementing an average linkage hierarchical clustering using the Jaccard distance as a metric in Matlab. Alternate clustering methods gave equivalent results. Rows were clustered with the same method, but, for Figs. 5A, C, and Fig. S3A, C, we built separate clustering trees for each class of interactions. In addition, to determine whether media that induce different types of interactions tend to spontaneously segregate, we applied the same clustering algorithm to the combined set of neutralism and mutualism-inducing media. We next counted the number of interactions of each type that were called correctly using the clustering. We obtained: TP = 10513; TN = 7964; FP = 2450; FN = 2468; Hypergeometric p-val = 0; accuracy (TP+TN)/(P+N) = 0.790 (T = true; F = false; P = positive (in this case, mutualism); N = negative (in this case, neutralism). The high accuracy suggests that it is possible to roughly discriminate mutualism and neutralism cases. However, this accuracy does not extend to the case of all four interaction types (data not shown).

### Robustness to environmental perturbations

For the *S. cerevisiae* and *E. coli* pair, 1000 media were chosen for each interaction class at random. For each of these media the set of metabolites was perturbed and the pair retested for interaction class 100 times. This was done by selecting a carbon containing metabolite, and replacing it with another carbon containing metabolite at random (but still allowing growth of the organisms in the joint model). The transition probabilities fore each interaction class were then calculated as the mean fraction of times a given interaction class is transformed into another interaction class upon the perturbation (Fig. 8A).

### Robustness analysis relative to perturbations of metabolic reactions

For the *S. cerevisiae* and *E. coli* pair, 1000 media were chosen at random. For each of these media, three types of reaction perturbations were performed 100 times. The first type of reaction perturbation consists of the deletion of a reaction at random from the joint model. The second type of reaction perturbation is implemented by adding a reaction at random to the joint model. The third type of perturbation corresponds to the simultaneous deletion of a reaction and addition of another reaction at random. The transition probabilities between any two interaction classes A and B were calculated as the mean fraction of times interaction class A became interaction class B after perturbation (Fig. 6A,B,C). In order to study the effects of multiple (*k*, ranging from 1 to 10) insults, we extended the perturbation analysis by randomly selecting and applying *k* random insertions or deletions 1000 times, and counting the number of times the interaction class change. The randomly added reactions were taken from the subset of KEGG reactions [70] involving metabolites present in the joint model.

## Supporting Information

Figure S1 Interaction-inducing media identified for the pair of yeast strains of Fig. 1B.
(0.11 MB PDF)

Figure S2 Results from the modeling of the syntrophic interaction between two S. cerevisiae strains engineered to be auxotrophic for adenine (Ade-) or lysine (Lys-) respectively.
(0.09 MB PDF)

Figure S3 Metabolite usage predicted for the pair of engineered yeast strains in media that induce commensal interactions.
(0.18 MB PDF)

Figure S4 Robustness of predicted interaction types upon gene deletions in the joint model for M. extorquens and M. barkeri.
(0.10 MB PDF)

Figure S5 Two examples depicting the details of environment-induced mutualistic interactions identified through the SIM algorithm for the (E. coli, M. barkeri) pair.
(0.15 MB PDF)

Figure S6 Interaction-inducing media identified for the S. cerevisiae - E. coli pair.
(0.14 MB PDF)

Table S1 Number of media that induce each possible class of interaction for every pair of microbes.
(0.01 MB XLS)

Table S2 List of metabolites composing the initial medium for applying the SIM algorithm to each pair of species.
(0.02 MB XLS)

Table S3 Possible inferences that one could make from comparing predicted outcomes of interactions during growth on different media with corresponding experimental results.
(0.01 MB XLS)

Table S4 Exchanged metabolites for two mutualism-inducing media for two different interacting pairs of organisms.
(0.01 MB XLS)

Text S1 Media ranking methods.
(0.06 MB PDF)
