# Supplementary material for: Environments that Induce Synthetic Microbial Ecosystems
Source: PLoS Comput Biol. 2010 Nov 18;6(11):e1001002. doi: 10.1371/journal.pcbi.1001002 (PMC2987903; doi:10.1371/journal.pcbi.1001002)
Supplement: Figure S1 — Interaction-inducing media identified for the pair of yeast strains of Fig. 1B. (0.11 MB PDF) [file pcbi.1001002.s001.pdf]

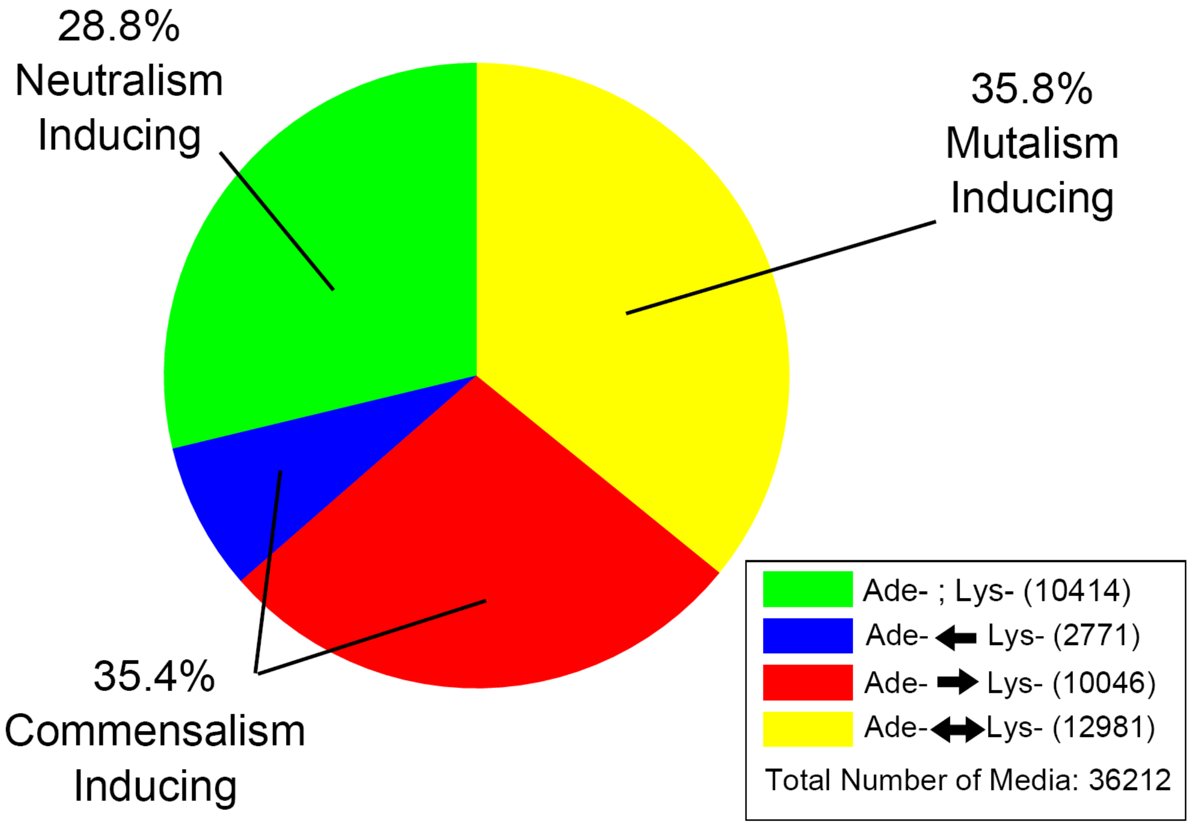

Figure S1

Interaction-inducing media identified for the engineered pair of yeast strains of Fig. 1B. Applying the SIM algorithm to the yeast pair identified a large number of interaction-inducing media. The size of each slice of the pie chart indicates the relative fraction of media which are identified to putatively induce each interaction class.
