# Supplementary material for: Environments that Induce Synthetic Microbial Ecosystems
Source: PLoS Comput Biol. 2010 Nov 18;6(11):e1001002. doi: 10.1371/journal.pcbi.1001002 (PMC2987903; doi:10.1371/journal.pcbi.1001002)
Supplement: Figure S2 — Results from the modeling of the syntrophic interaction between two S. cerevisiae strains engineered to be auxotrophic for adenine (Ade-) or lysine (Lys-) respectively. (0.09 MB PDF) [file pcbi.1001002.s002.pdf]

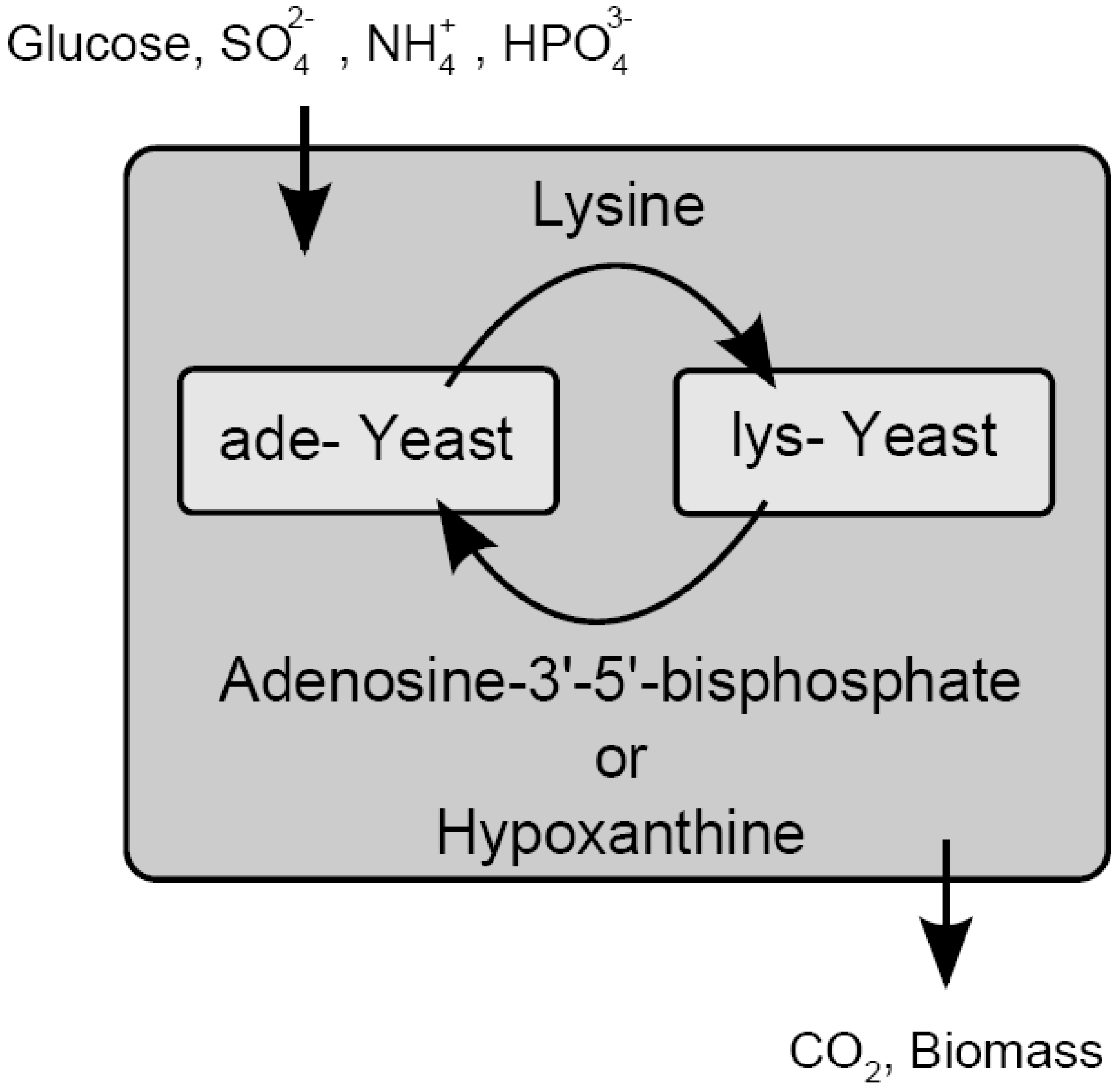

Figure S2

Results from the modeling of the syntrophic interaction between two *S. cerevisiae* strains engineered to be auxotrophic for adenine (Ade-) or lysine (Lys-) respectively.

We simulated this system building a joint stoichiometric models of two yeast strains modified to reflect these auxotrophies. To verify that this joint model was working correctly, we tested for simulated growth with and without exchange of metabolites between the two organisms. When exchange of metabolites is not allowed, no growth is possible under a minimal glucose medium. Conversely, when we allow the exchange of all extracellular metabolites, growth is possible for both organisms. By applying our "search for exchanged metabolites" (SEM) algorithm to this engineered yeast pair, we found that the organisms should be expected to exchange lysine, and either of two adenine derivatives, hypoxanthine (HXAN) and adenosine-3'-5'-bisphosphate (PAP). These results

can be explained by combining metabolic network connectivity with feasibility and directionality of extracellular transport (see Fig. 4 for more details). It had been previously shown that both HXAN (as in our model) and inosine (in contrast to our model) can be exported in yeast [1,2]. We could not find any report of direct evidence for the efflux of either Ade or PAP in yeast. While in the Shou *et al.* paper [3], adenine is proposed as a likely exchanged metabolite, this seems to be inferred from an indirect method of measurement. Thus their results would be explained equally as well if the exported metabolite was another pyrimidine such as HXAN (or inosine), as predicted by our model. These new predictions are amenable to experimental testing.

1. Ullman B, Kaur K, Watts T (1983) Genetic studies on the role of the nucleoside transport function in nucleoside efflux, the inosine cycle, and purine biosynthesis. Mol. Cell. Biol. 3: 1187-1196.
2. Lecoq K, Konrad M, Daignan-Fornier B (2000) Yeast GMP Kinase Mutants Constitutively Express AMP Biosynthesis Genes by Phenocopying a Hypoxanthine-Guanine Phosphoribosyltransferase Defect. Genetics 156: 953-961.
3. Shou W, Ram S, Vilar JMG (2007) Synthetic cooperation in engineered yeast populations. Proc Natl Acad Sci U S A 104: 1877-82.
