# Supplementary material for: Environments that Induce Synthetic Microbial Ecosystems
Source: PLoS Comput Biol. 2010 Nov 18;6(11):e1001002. doi: 10.1371/journal.pcbi.1001002 (PMC2987903; doi:10.1371/journal.pcbi.1001002)
Supplement: Figure S3 — Metabolite usage predicted for the pair of engineered yeast strains in media that induce commensal interactions. (0.18 MB PDF) [file pcbi.1001002.s003.pdf]

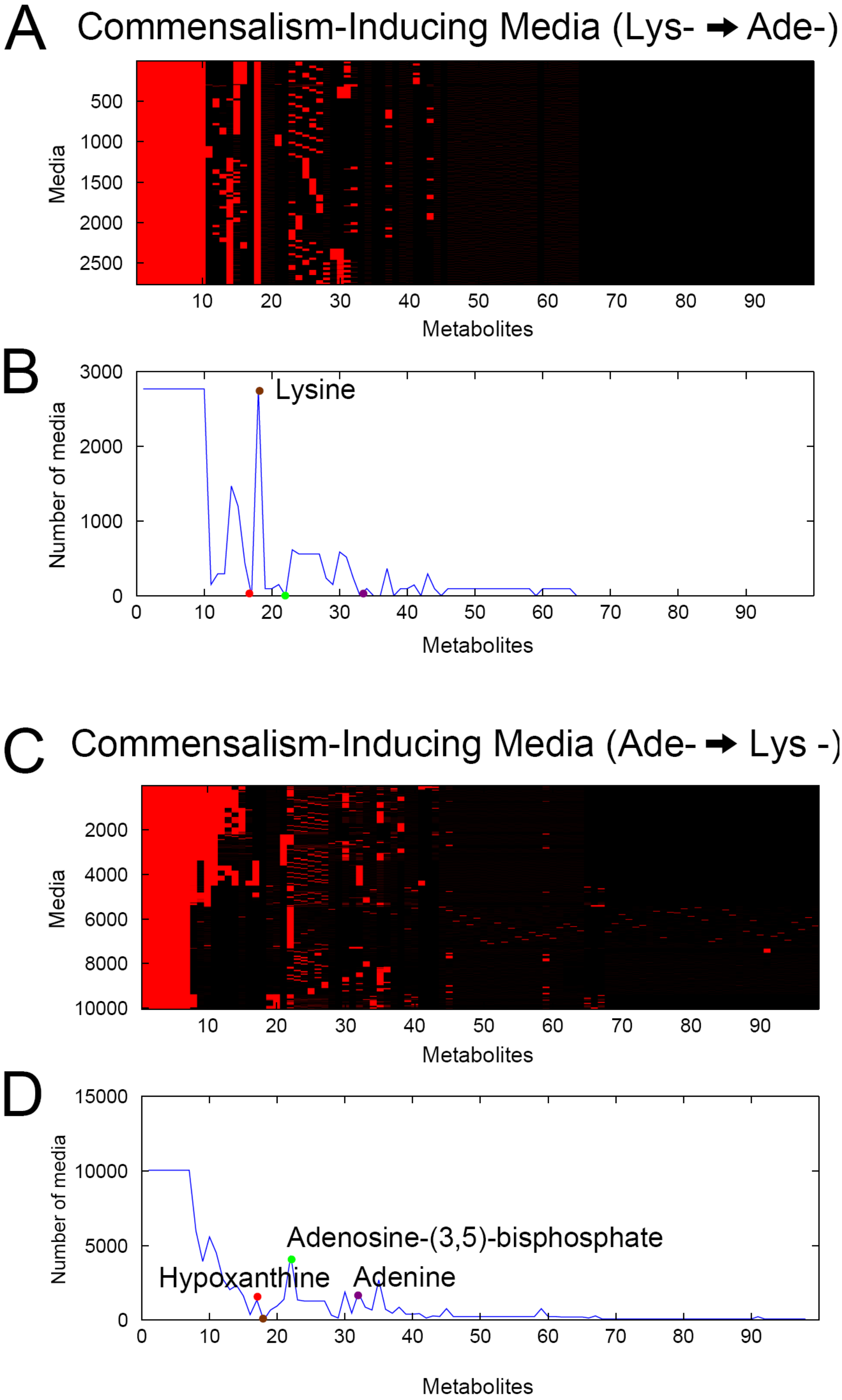

A
Commensalism-Inducing Media (Lys- ➡ Ade-)
Media
500
1000
1500
2000
2500
10
20
30
40
50
60
70
80
90
Metabolites
B
Number of media
3000
2000
1000
0
Lysine
0
10
20
30
40
50
60
70
80
90
Metabolites
C
Commensalism-Inducing Media (Ade- ➡ Lys -)
Media
2000
4000
6000
8000
10000
10
20
30
40
50
60
70
80
90
Metabolites
D
Number of media
15000
10000
5000
0
Adenosine-(3,5)-bisphosphate
Hypoxanthine
Adenine
0
10
20
30
40
50
60
70
80
90
Metabolites

Figure S3

Metabolite usage predicted for the pair of engineered yeast strains in media that induce commensal interactions. Metabolites in all panels of this figure are clustered according to their presence in all media analyzed (see Methods).

A Binary map of metabolite usage (black = not used; red = used) across all (Lys- ⇨ Ade-) commensal- inducing media. A large set of metabolites is used in different combinations, forming a complex map of mutual capacities to compensate each other.

B By looking at the overall frequency of metabolite usage across all (Lys- ⇨ Ade-) commensalism-inducing media, one can see that lysine is always present, as expected, while adenine derivative metabolites are absent.

C The binary map (as in A) for all (Ade- ⇨ Lys-) commensal-inducing media.

D Frequency of metabolites across all (Ade- ⇨ Lys-) commensalism-inducing media. These media never contain the lysine, but do contain many adenine derivatives as is expected.
