# Supplementary material for: Environments that Induce Synthetic Microbial Ecosystems
Source: PLoS Comput Biol. 2010 Nov 18;6(11):e1001002. doi: 10.1371/journal.pcbi.1001002 (PMC2987903; doi:10.1371/journal.pcbi.1001002)
Supplement: Figure S4 — Robustness of predicted interaction types upon gene deletions in the joint model for M. extorquens and M. barkeri. (0.10 MB PDF) [file pcbi.1001002.s004.pdf]

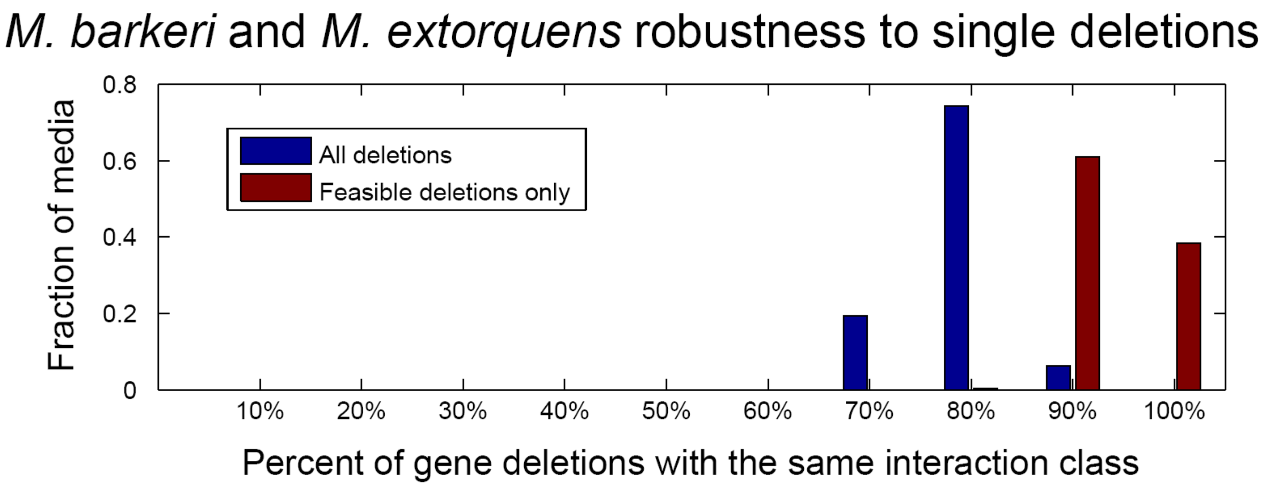

Figure S4

Robustness of predicted interaction types upon gene deletions in the joint model for *M. extorquens* and *M. barkeri*. In this analysis, each metabolic reaction in the joint models of *M. extorquens* and *M. barkeri* was knocked out (by setting $v_i = 0$) iteratively. We then re-tested each medium we had previously identified to determine the interaction class of the mutant. For each medium we compute the percent of knockouts for which the interaction type (see Table 1) is the same as without the knockout. We then bin all these percentages for the different media (in bins of 10% intervals), and normalize to the total number of media, to give rise to the above histogram. Hence, this graph shows the fraction of media that have a given percent interaction consistency across all single deletions. This value is determined for all media and knockouts (in blue), as well as for the subset of media and knockout cases that did not give rise to infeasibility in the LP problem (in red). The results of this analysis show that the identification of interaction classes is robust relative to perturbations of the initial stoichiometric model (see also Fig. 6)
