# Supplementary material for: Environments that Induce Synthetic Microbial Ecosystems
Source: PLoS Comput Biol. 2010 Nov 18;6(11):e1001002. doi: 10.1371/journal.pcbi.1001002 (PMC2987903; doi:10.1371/journal.pcbi.1001002)
Supplement: Figure S5 — Two examples depicting the details of environment-induced mutualistic interactions identified through the SIM algorithm for the (E. coli, M. barkeri) pair. (0.15 MB PDF) [file pcbi.1001002.s005.pdf]

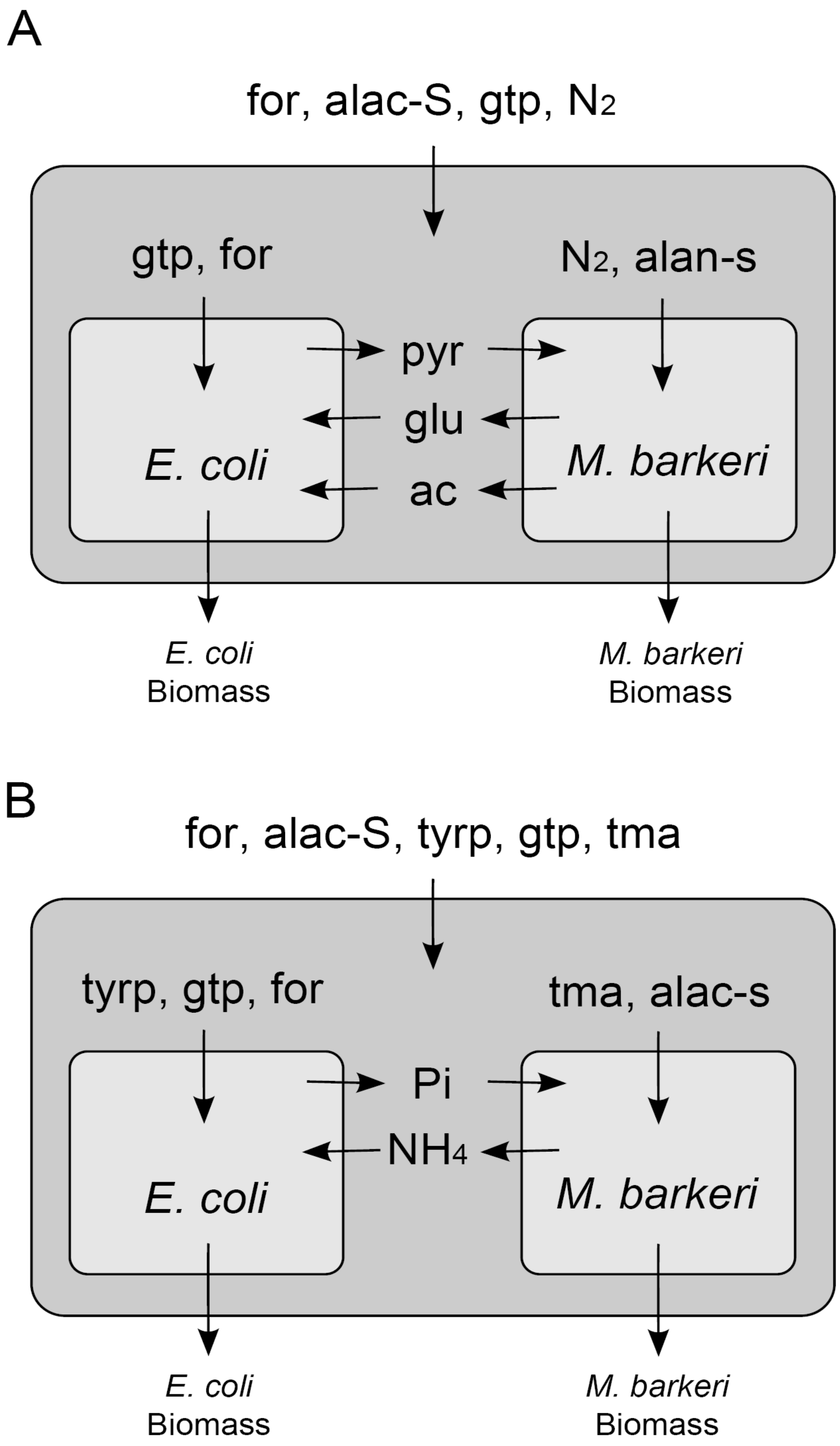

A
for, alac-S, gtp, N2
gtp, for
N2, alan-s
pyr
glu
ac
E. coli
M. barkeri
E. coli Biomass
M. barkeri Biomass
B
for, alac-S, tyrp, gtp, tma
tyrp, gtp, for
tma, alac-s
Pi
NH4
E. coli
M. barkeri
E. coli Biomass
M. barkeri Biomass

Figure S5

Two examples depicting the details of environment-induced mutualistic interactions identified through the SIM algorithm for the *E. coli - M. barkeri* pair. The two media, selected here based on high *F* score ranking (see Supplementary Methods), are among the ones that give rise to mutualistic interactions.

A In the first example *M. barkeri* appears to be responsible for fixing nitrogen into a form *E.coli* can use, glutamate in this case. Curiously, even though *M.barkeri* is able to access the acytl-lactate in the medium, this appears to be insufficient to support its growth. An energy rich carbon, in the form of pyruvate, is provided to *M.barkeri* by *E.coli.*

B In our second example, while the medium is more complex, the exchange of metabolites is more straightforward. *E. coli* utilizes a number of phosphorus containing metabolites and creates inorganic phosphate that *M. barkeri* can use. In a complementary fashion, *M. barkeri* metabolizes a number of nitrogen containing compounds and release excess ammonium that *E. coli* can use.
