# Supplementary material for: Environments that Induce Synthetic Microbial Ecosystems
Source: PLoS Comput Biol. 2010 Nov 18;6(11):e1001002. doi: 10.1371/journal.pcbi.1001002 (PMC2987903; doi:10.1371/journal.pcbi.1001002)
Supplement: Figure S6 — Interaction-inducing media identified for the S. cerevisiae - E. coli pair. (0.14 MB PDF) [file pcbi.1001002.s006.pdf]

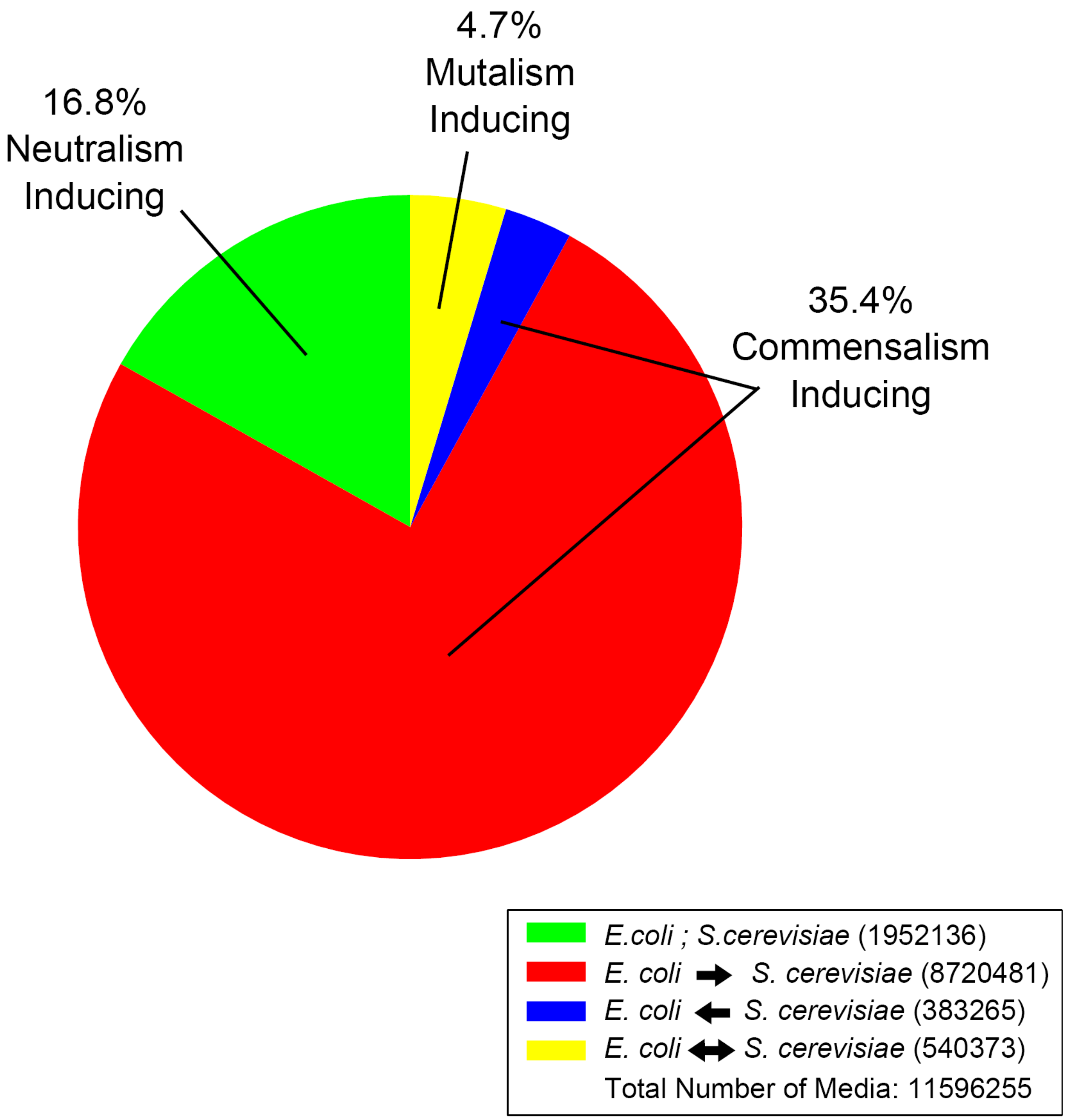

Figure S6

Interaction-inducing media identified for the *S. cerevisiae* - *E. coli* pair. Applying the SIM algorithm to the yeast - *E. coli* pair identified a very large number of interaction-inducing media. The relative size of each slice of the pie chart indicates the fraction of media identified for each induced interaction class.
