# Supplementary material for: Environments that Induce Synthetic Microbial Ecosystems
Source: PLoS Comput Biol. 2010 Nov 18;6(11):e1001002. doi: 10.1371/journal.pcbi.1001002 (PMC2987903; doi:10.1371/journal.pcbi.1001002)
Supplement: Text S1 — Media ranking methods. (0.06 MB PDF) [file pcbi.1001002.s011.pdf]

Supplementary Text S1

Media ranking

Our algorithm for the search of interaction-inducing media (SIM) may produce a large set of solutions. In order to select specific media both for further computational analyses and for experimental testing it is useful to develop possible criteria for ranking such solutions. Here we propose a simple strategy for selection pairs to test.

Method 1

It stands to reason that those metabolites which appear more frequently in our predicted media sets, may have a greater chance of success. This method therefor identifies, for a specific interaction-inducing pair, those media which have the most commonly occurring metabolites across all predicted media. The first step in this process is to estimate the fraction of media in which each metabolite is found. If Ω is the set of all identified media, M denotes a specific medium, and m a specific metabolite, then the fraction of media containing m can be expressed as:

$$f(m) = |\{M \in \Omega \text{ s.t. } m \in M\}| / |\Omega|$$

The second step is to compute, for each medium M, a score based on its metabolite content. Each metabolite m contributes multiplicatively to this score with its total fraction f(m), in an expression that may be thought of as analogous to a likelihood of the medium:

$$F(M) = \Pi_{m \in M} [f(m)]$$

The scoring function F(M) can be used to rank media, with large F indicating a medium that contains the most common metabolites.

Method 2

An alternate method of ranking media is to consider the number of reactions each metabolite participates in, within the metabolic network of the pair of organisms. A metabolite with a large number of reactions in a particular model is more likely to be useable; hence a medium could be considered more likely to induce the expected interaction if it contains metabolites involved in many reactions. This ranking can be implemented with a small modification of the above definitions. If R denotes the set of metabolites that participate in a given reaction, and m a specific metabolite, then the fraction of reactions containing m can be expressed as:

$$g(m) = |\{R \in \Psi \text{ s.t. } m \in R\}| / |\Psi|$$

As above, for each medium M, a score based on its metabolite content, can be computed as:

$$G(M) = \Pi_{m \in M} [g(m)] .$$
